# A call for doubling the diagnostic rate of at-risk metabolic dysfunction-associated steatohepatitis

**DOI:** 10.1016/j.lanepe.2025.101320

**Published:** 2025-06-04

**Authors:** Jeffrey V. Lazarus, Paul N. Brennan, Henry E. Mark, William Alazawi, Alina M. Allen, Christopher D. Byrne, Laurent Castera, Cyrielle Caussy, Kenneth Cusi, Martin M. Grajower, Christopher J. Kopka, Jo Massoels, Michael Roden, C Wendy Spearman, Frank Tacke, Vincent Wai-Sun Wong, Mazen Noureddin

**Affiliations:** a City University of New York Graduate School of Public Health and Health Policy (CUNY SPH), New York, NY, USA; b Barcelona Institute for Global Health (ISGlobal), University of Barcelona, Barcelona, Spain; c Faculty of Medicine and Health Sciences, University of Barcelona (UB), Barcelona, Spain; d Division of Molecular and Clinical Medicine, Ninewells Hospital and Medical School, University of Dundee, Dundee, UK; e Barts Liver Centre, Blizard Institute, Queen Mary University of London, London, UK; f Division of Gastroenterology and Hepatology, Department of Medicine, Mayo Clinic, Rochester, MN, USA; g Southampton National Institute for Health and Care Research, Biomedical Research Centre, University Hospital Southampton and University of Southampton, Southampton, UK; h Université Paris Cité, Department of Hepatology, Hospital Beaujon, AP-HP, Clichy, Paris, France; i Endocrinology Diabetes Nutrition, Hôpital Lyon Sud, Hospices Civils de Lyon, Lyon, France; j Division of Endocrinology, Diabetes & Metabolism, Department of Medicine, University of Florida, Gainesville, FL, USA; k Division of Endocrinology, Department of Medicine, Albert Einstein College of Medicine, Bronx, NY, USA; l Echosens, Paris, France; m Department of Endocrinology and Diabetology, Medical Faculty and University Hospital Düsseldorf, Heinrich Heine University, Düsseldorf, Germany; n Institute for Clinical Diabetology, German Diabetes Center (DDZ), Leibniz Center for Diabetes Research at Heinrich Heine University, Düsseldorf, Germany; o Division of Hepatology, Department of Medicine, Faculty of Health Sciences, University of Cape Town, Cape Town, South Africa; p Department of Hepatology & Gastroenterology, Charité - Universitätsmedizin Berlin, Berlin, Germany; q Department of Medicine and Therapeutics, The Chinese University of Hong Kong, Hong Kong; r Houston Methodist Hospital, Houston, TX, USA; s Houston Research Institute, Houston, TX, USA

**Keywords:** MASLD, MASH, Diagnostic rate, Health policy, Health systems, Public health

## Abstract

Metabolic dysfunction-associated steatohepatitis (MASH) is an increasingly important contributor to morbidity and mortality. Little emphasis has been placed on its timely diagnosis and interventions to prevent adverse disease outcomes. The principal determinant of MASH outcomes is the liver fibrosis stage. The prevalence of MASH is higher among people living with obesity and/or type 2 diabetes, with MASH with moderate to advanced fibrosis affecting one in six adults. Delivering a paradigm shift in MASH diagnosis in the four countries studied will require an expansion of community-based diagnostic capability that will also foster prevention efforts and provide opportunities for treatment and care.

**Search strategy and selection criteria**

References for this article were identified through PubMed, Google Scholar, and Internet searches, with no time restriction. The articles, guidelines, reports, and policies found in English were screened for inclusion of liver health, and specifically metabolic dysfunction-associated steatotic liver disease (MASLD). Relevant documents were reviewed to identify the strategies, approaches, diagnostic tools, and pathways employed in populations at-risk for MASLD and metabolic dysfunction-associated steatohepatitis, including people living with common comorbidities. The final reference list was generated on the basis of originality and relevance to the broad scope of this article.

## Introduction

An estimated 32.4% (95% CI: 29.9–34.9) of adults globally have metabolic dysfunction-associated steatotic liver disease (MASLD, formerly NAFLD),^1^ with regional prevalence ranging from 32.6% (24.5–40.6) in Europe to 44.8% (25.9–99.7) in North America.^2^ The prevalence of metabolic dysfunction-associated steatohepatitis (MASH, formerly NASH),^1^ the more aggressive form of MASLD, is commonly reported as 5%.^3^ However, there are large variations in the contextualisation of the epidemiological data along with geographic and patient-specific factors. The disease exists across a spectrum of fibrosis severity, commonly classified across 5 stages from F0 (no fibrosis) to F4 (cirrhosis).^4^ Fibrosis stage is the most important prognostic marker for people living with MASLD.^5^ Fibrosis stage ≥F2 is commonly referred to as ‘moderate fibrosis’, and ≥F3 as ‘advanced fibrosis’, while MASH combined with F2 or greater is known as ‘at-risk MASH’. ‘At-risk MASH’ is a term of reference to describe those living with MASLD and the risk of fibrosis, and is more comprehensively covered in the EASL-EASD-EASO clinical practice guideline from 2024.^6^

Hepatic steatosis shares a complex bidirectional relationship with components of metabolic syndrome and is strongly, but not exclusively, associated with obesity and insulin resistance.^7^^,^^8^ MASH with fibrosis F2 or greater is much more prevalent in adults living with type 2 diabetes (T2D) than in the general adult population and is found in one in six older adults (≥50 years).^9^^,^^10^ MASLD is associated with the risk of developing extra-hepatic diseases, including cardiovascular disease (CVD)–the leading cause of death in people living with the condition^11^–T2D, chronic kidney disease, and extra-hepatic cancer,^12^, ^13^, ^14^ with the risk being proportional to the severity of the disease.^14^ The burden of hepatic and non-hepatic morbidity and mortality related to MASH is large and growing,^15^^,^^16^ presenting a major challenge for health systems.^17^ An important aspect of care pertains to increasing awareness around MASLD and co-ordinating metabolic risk optimisation in patients living with the condition across the continuum of metabolic-dysfunction.^18^ The development of cross-specialist, multidisciplinary teams and integrated clinical pathways to optimise MASLD and MASH care are a great unmet need. Additionally, non-pharmacological interventions (NPIs) are the cornerstone of improved health outcomes in people living with these conditions and should be centrally positioned alongside targeted pharmacological approaches, particularly as liver-specific and pleiotropic treatments become available.^19^

Despite the scale of the challenge, MASLD is underdiagnosed among the general population and in specialist care settings including cardiology, endocrinology (diabetology specifically), and metabolic medicine.^20^ The largely asymptomatic nature of the disease, including at the most advanced stages, often results in patients presenting with decompensated cirrhosis or advanced hepatocellular carcinoma (HCC),^21^ with the burden of MASH-associated primary liver cancer having increased over the past 20 years.^22^ Each late-stage MASH diagnosis represents a missed opportunity for earlier intervention to prevent disease progression, threatening worse hepatic and extra-hepatic outcomes for people living with the condition and greater costs for individuals, health systems, and societies.^23^, ^24^, ^25^

In 2023, a global collaboration of over 300 experts and practitioners^26^ called for the development of clear guidance on care pathways that promote the timely referral of people living with MASLD and MASH and for cross-disciplinary work to establish the most efficient and effective means of identifying people at risk. Some clinical guidelines include pathways and algorithms for identifying advanced disease in non-hepatology settings,^6^ yet the operational readiness to implement these is low in many countries. Challenges include how to absorb the intensifying need for diagnostics along with treatment and care, and overcoming the limited capacity for task-shifting diagnostic performance outside of hepatology clinics. In addition, there is a low perceived readiness across most health systems pertaining to the rapid integration and implementation of new diagnostics.^27^

There is, however, cause for optimism. Advances in non-invasive diagnostic capabilities can facilitate a shift from specialist-only diagnosis (e.g. liver biopsy) to generalist diagnosis (e.g. non-invasive tests (NITs) at the primary care level). Artificial intelligence-supported automation for liver function screening, often from pre-existing data within electronic health records,^28^^,^^29^ offers the near-term possibility of heightened productivity and task-shifting. Alongside these diagnostic advances to improve case finding is an ever-growing toolkit for treatment and care, including the first pharmacological therapy for MASH (resmetirom), approved by the US Food and Drug Administration (FDA) in March 2024,^30^ the expectance of approval in 2025 of semaglutide, based on the recent phase 3 ESSENCE trial,^31^ and more treatments expected to become available within the next 1–3 years.^32^^,^^33^ There is evidence, for example, of MASH histological benefits from four classes of pharmacological treatments, some of which are used for obesity and/or T2D, including peroxisome proliferator-activated receptor agonists,^34^ glucagon-like peptide-1 receptor (GLP-1R) agonists,^35^ dual glucose-dependent insulinotropic polypeptide-GLP-1R agonists,^36^ and triple agonists,^37^^,^^38^ though none are currently approved as MASH specific treatments. Such landmark moments in MASLD and MASH treatment may create an upstream benefit in catalysing expanded diagnostics, hopefully improving outcomes for the millions living with the disease.

In this paper, we outline a realistic near-term strategic approach to deliver a paradigm shift in MASH diagnosis before 2030, a date chosen to align with the United Nations’ Sustainable Development Goals,^39^ with an emphasis on efforts to ensure healthy lives and promote well-being for all at every age.

## The MASLD and MASH diagnosis dilemma–who, what, and where

MASLD regression is most feasible at the earliest stages of fibrosis (i.e. F0-1), in some instances exclusively with NPIs,^40^ so a strong case can be made for diagnosing at the earliest opportunity. In the short term, capacity and reimbursement constraints within health systems have led to a more targeted approach focused on identifying those who are at greatest risk of nearer-term adverse outcomes, and so likely to experience improved quality of life and reduced risk of premature mortality following a diagnosis and linkage to care.

This raises three critical questions: Who are the highest priority groups to identify? What is the most cost-effective means of identifying them? And where are the opportunities to identify these groups within the health system?

### Who are the highest priority groups to identify and refer to specialist care?

People living with MASH (either presumed based on NITs or confirmed by liver biopsy) who also have moderate fibrosis (≥F2, i.e. people with at-risk MASH) are a particular interest group for hepatologists and gastroenterologists, with care focused on preventing fibrosis progression to cirrhosis and HCC.^8^ The incidence of HCC in those living with MASLD without cirrhosis may be as high as 35%,^41^ and therefore efforts to minimise the development of fibrogenesis is pivotal in mitigating against this. Evidence and guidance from international societies do not advocate for hepatoma surveillance programmes; however, in the future, novel approaches using risk stratification based on NITs may be implemented. Further research is needed to establish criteria for HCC surveillance based on a liver stiffness measurement (LSM) and other clinical criteria. As the numbers of these subjects is so great, magnetic resonance elastography, or traditional histological approaches, will not be feasible for this purpose.

Pharmacological trials have and continue to focus on this group of at-risk MASH, with resmetirom being indicated for use in people living with MASH (with no liver biopsy confirmation required) and fibrosis stage 2 or 3.^42^

While people living with at-risk MASH represent a small proportion of the overall population living with MASLD, in absolute terms it is a large cohort of people who would benefit from being linked to care with a hepatologist or gastroenterologist.^3^

The lack of specific symptoms arising from MASLD, even in the more advanced stages, requires a risk-based approach to population enhancement and positive case-identification. Obesity, pre-diabetes, and T2D are all independent risk factors for MASH.^43^

Our recommendation is to focus screening and active case finding efforts on people living with any of the following: 1) T2D; 2) obesity and one or more cardiometabolic risk factor(s) (e.g. dyslipidaemia, hypertension, and pre-diabetes); and 3) persistently elevated liver enzymes (over a period longer than six months, when tested more than four weeks apart). This is a pragmatic and strategic choice, with well-defined groups at elevated risk of MASH and advanced fibrosis^44^ and in accordance with multidisciplinary European guidelines.^6^

### What is the most cost-effective approach to identify people with MASH and advanced fibrosis?

The advent of NITs has allowed clinical practice to largely move beyond the biopsy, which is a costly and invasive procedure that carries a degree of risk.^45^ NITs are separated into two broad categories: 1) biomarkers in serum samples; and 2) LSMs using ultrasound–or magnetic resonance–based elastography techniques.^46^ A range of NITs are used in clinical practice, from non-proprietary calculated indices (e.g. FIB-4) to proprietary serum-based and elastography-based tests. The availability of different NITs varies widely across healthcare settings.

The practical application of NITs involves setting pre-determined cut-offs related to the risk of moderate or advanced liver fibrosis. A low cut-off improves the sensitivity and results in a high negative predictive value. When used in the general population (i.e. a low prevalence cohort) clinicians can rule out advanced fibrosis when a result falls below the cut-off. Conversely, a high cut-off improves the specificity and positive predictive value and ability to rule in advanced fibrosis.^47^^,^^48^ While current NITs are not as accurate for detecting ≥F2 as they are for ≥F3, they are still reliable enough for use in routine clinical care. Clinicians often repeat the same test over time or use different NITs sequentially (stepwise), balancing cost and availability; both approaches are expected to increase diagnostic performance.^45^ The performance of individual tests also varies in different population groups; perhaps most importantly in people living with T2D, where overall performance is often poorer than in individuals living without T2D.^49^

The cost-effectiveness of screening and active case finding approaches has been hotly debated. The models are influenced by the inclusion of extrahepatic outcomes, which generally occur prior to hepatic outcomes and can be costly.^50^, ^51^, ^52^ A 2024 cost-effectiveness study on screening for clinically suspected MASLD in people living with T2D and obesity, with multiple cardiometabolic risk factors, found favourable incremental cost-effectiveness ratios.^52^

Critically, as pharmacological–liver- and weight loss-directed–and non-pharmacological approaches become more efficacious and available, the cost-effectiveness of active case finding may become substantially more favourable,^53^ including early-stage diagnosis to promote prevention of disease progression.

### Where are the opportunities to identify people living with MASH and advanced fibrosis within the health system?

**Fig. 1:**
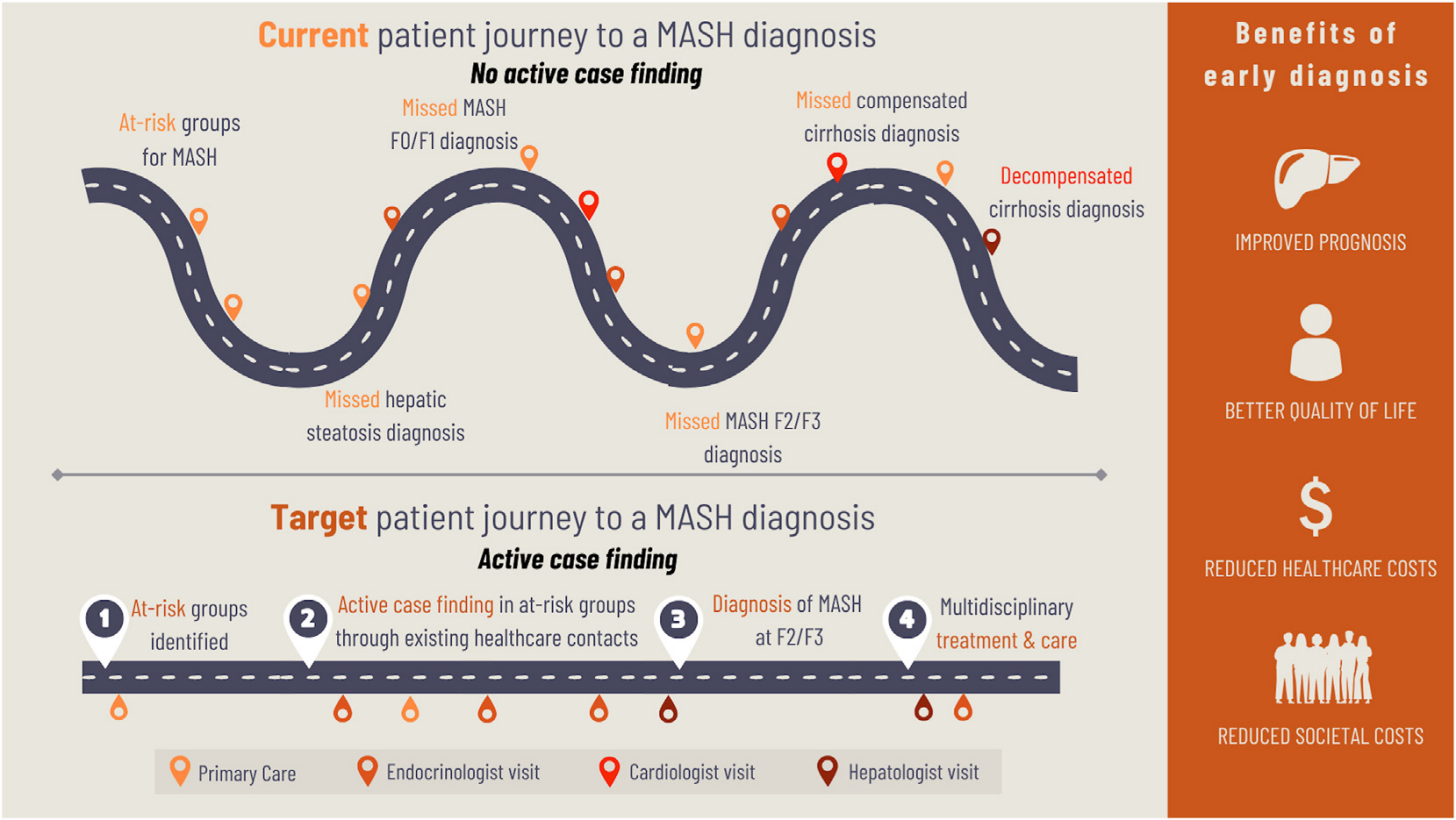
Current and target pathways to a MASH diagnosis. Abbreviations: MASH, metabolic dysfunction-associated steatohepatitis.

People living with T2D alone or cardiometabolic multimorbidity tend to have a high number of healthcare contacts annually in primary and secondary care (e.g. with endocrinologists/diabetologists).^54^ Each healthcare contact represents a potential opportunity to assess for at-risk MASH in people living with obesity and pre-diabetes or diabetes. Incidental detection of hepatic steatosis by radiologists when imaging for other conditions, for example, also presents an opportunity and pathways should be in place to enable fibrosis risk assessment following such a finding. Today, most of these opportunities are overlooked (Fig. 1).

Most of these healthcare visits occur outside of specialised liver clinics, requiring a broad expansion of testing in other areas of the health system. Capacity constraints among hepatologists and gastroenterologists preclude the option of first-line tests, or many second-line tests, being undertaken exclusively in hepatology and gastroenterology settings. While endocrinology, cardiology, and primary care experience similar resource constraints, the pre-existing healthcare contacts in these clinical settings, including many who undergo blood or imaging tests, make these visits amenable to first-tier assessment of liver fibrosis. We suggest that such integration will be more efficient, cost-effective, and person-centric by minimising the additional visit burden on individuals. Barriers to shifting diagnostics beyond hepatology and gastroenterology should not be underestimated and will require a broad expansion of the MASLD community of practice, to address current under- and late-diagnosis challenges, and the delivery of multidisciplinary care models.^55^

Expanding the MASLD community of practice is a visionary and practical endeavour. Among healthcare professionals, this is driven by an understanding of who needs to be engaged, what the benefit of engaging is for their patients, and what is being asked of them (e.g. deliver diagnostic tests, make referrals for testing). At least initially, the target should be primary care doctors, endocrinologists/diabetologists, cardiologists, and obesity specialists, along with allied health professionals who work across these disciplines. Of equal importance is engagement with people living with MASLD and patient advocates as a powerful voice in calling for equitable access to diagnostics, treatment, and care and in creating demand for diagnostic testing. This will include groups focused on diabetes, obesity, heart disease, cancer, and non-communicable diseases more generally.

## Translating the promise of innovation at scale

Increasing MASH diagnoses will rely on innovation across a range of areas, from technological advances in diagnostic hardware and software to artificial intelligence (AI) supported task-shifting and innovation in care models.

By leveraging existing technologies, in the next three years we can feasibly advocate for incorporation of the FIB-4 test and care pathway algorithms into electronic medical records and automation of FIB-4 and other serum based fibrosis tests^56^–such as the Enhanced Liver Fibrosis (ELF) test, which has FDA marketing authorisation for prognostication of disease progression but is presently not widely available or used–within routine clinical laboratory results for target individuals. In the UK, ELF is recommended as the first biomarker test of choice in the 2016 NICE NAFLD Guideline,^57^ although its use is restricted to certain centres that have approved funding for utilisation within primary and secondary healthcare. As a serum-based biomarker, there is a clear advantage to implementation of ELF reflex testing following a positive or indeterminate index test, like FIB-4. The development of simple guidance for primary care providers will be key to delivering this at pace and scale. In some health systems this drive needs to be supported by patient group advocacy and reimbursement incentives for active case finding of at-risk MASH, with the goal of making the assessment as routine as that of cardiovascular risk and blood pressure, glycated haemoglobin, or cholesterol levels.

Some relatively simple actions can also be taken now. In addition to broad implementation of screening based on current MASLD guidelines of FIB-4 ± vibration controlled transient elastography-LSM,^6^ several clinical guidelines suggest levels for liver enzymes above which further investigation is required. For instance, the American Association of Clinical Endocrinology suggests an alanine aminotransferase (ALT) level of >30 U/L^58^ and the American College of Gastroenterology an ALT >33 IU/L for males and >25 IU/L for females.^59^ Yet, many laboratories use ALT cut-offs of 40–45 U/L or higher, especially where some outdated analytical techniques are employed; meanwhile, the International Federation of Clinical Chemistry recommends an ALT threshold of 42 U/L for males and 30 U/L for females, using modern techniques.^60^ Important first steps would include lowering the ‘normal’ laboratory values for liver enzymes to be in line with clinical practice guidelines and linking identified individuals to confirmatory testing. Equally, introducing a quality measure, such as an annual liver fibrosis assessment, into diabetes and obesity management would be a notable advance for the field.

Within three years, the availability of second-line testing (e.g. liver elastography or blood-based tests) should be widely expanded to facilitate referral pathways from primary care more efficiently; the success of this will also depend on diagnostic tools being accessible and affordable, including through adequate reimbursement policies. Existing diagnostic pathways published in clinical practice guidelines should be used according to the context and available resources, acknowledging that they may perform variably across different populations.

Within 3–5 years, the field should position itself for technology-based step changes. Although guidelines advocate for unified approaches, there is no definitive consensus on how to deliver this paradigm change. Advances in AI that are already supporting liver function test task-shifting in some contexts are likely to expand to other geographic regions,^61^ with machine learning also enhancing productivity and predictive accuracy.^62^ Innovations in diagnostics will facilitate quicker, more accurate, and easier-to-use tools. Alongside this, a growing treatment toolkit will drive greater demand for MASH diagnosis among people living with the disease, which should lead to a broadening of testing within and across healthcare settings. This shift should also facilitate much earlier diagnosis, allowing for a greater focus on prevention through the full spectrum of MASLD, including individuals without fibrosis; such “preventive hepatology” would represent a second stage of the paradigm shift in MASLD diagnosis, treatment, and care and simultaneously contribute to better metabolic health overall (Box 1).

Box 1 **Doubling the diagnosis rate of at-risk MASH by 2027–an achievable aspiration or a bridge too far?**

**Fig. 2:**
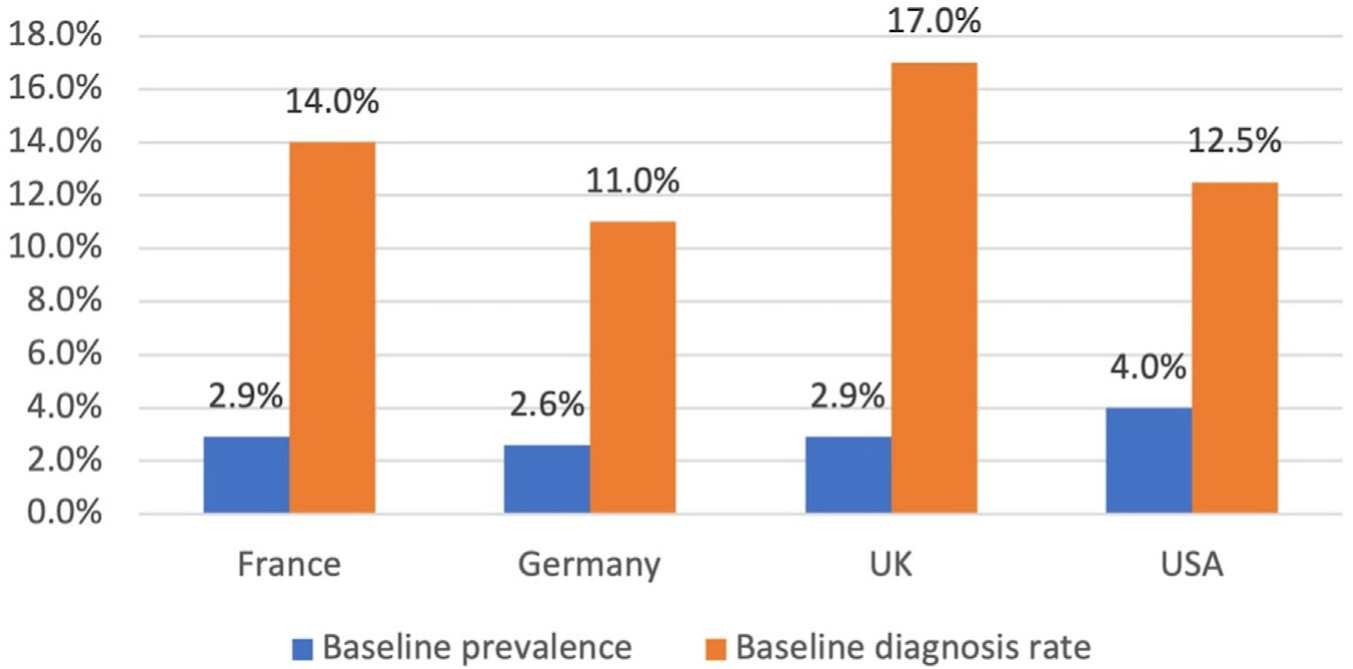
The estimated ≥F2 metabolic dysfunction-associated steatohepatitis prevalence in France, Germany, the UK, and the USA, and the proportion diagnosed of this population, in 2021.

**Fig. 3:**
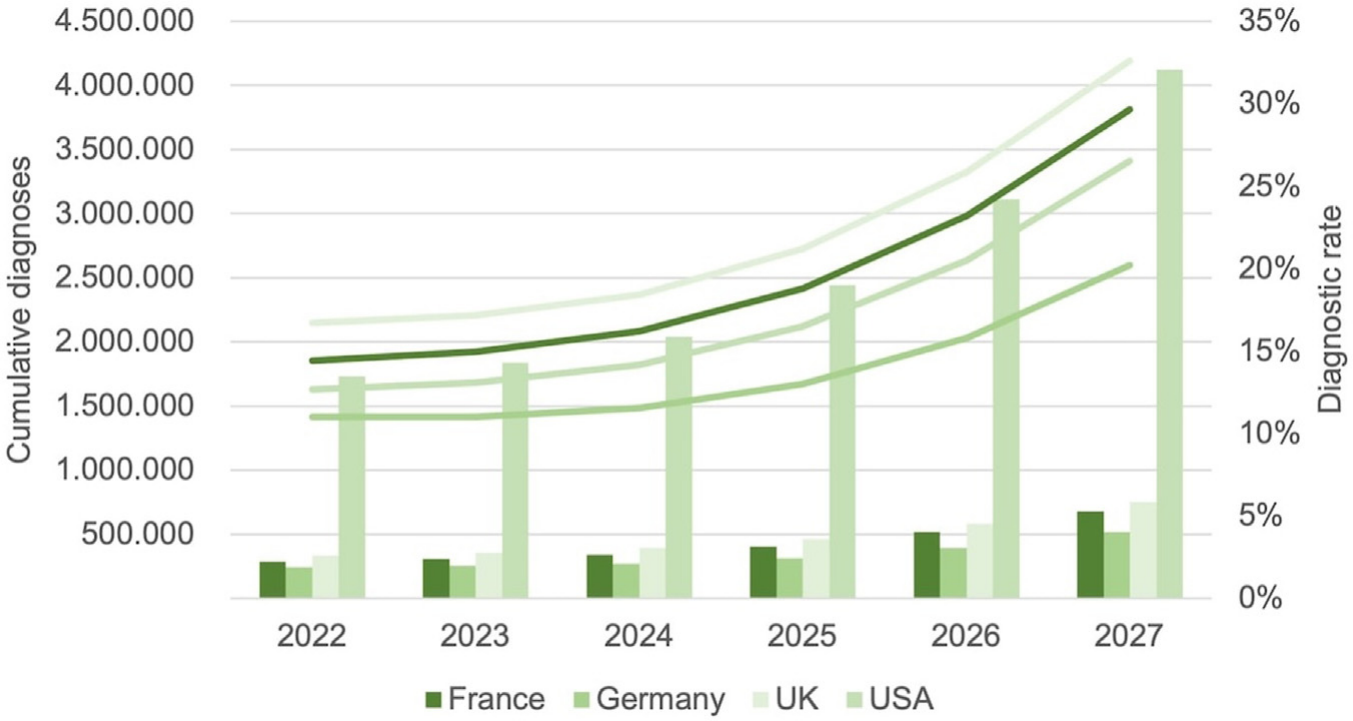
Model extrapolating cumulative diagnoses (absolute) and diagnostic rate (%) from 2022 to 2027 in France, Germany, the UK, and the USA, assuming testing expansions to non-hepatology/gastroenterology settings.

In the absence of globally agreed targets for addressing the burden of metabolic dysfunction-associated steatotic liver disease (MASLD), such as those that could be provided by the World Health Organization, as an interim goal we considered how feasible it would be to double the at-risk metabolic dysfunction-associated steatohepatitis (MASH) diagnosis rate (i.e. MASH with F2 or F3) within 5 years. The doubling target was chosen as a major yet realistic initial step towards a meaningful increase that gets us closer to the expected prevalence. For this purpose, we estimated the testing requirements to double the diagnosis rate of at-risk MASH between 2022 and 2027 in France, Germany, the UK, and the USA. The prevalence of ≥F2 MASH in the general population and diagnostic rates as of 2021 (Fig. 2) were estimated based on the triangulation of published^63^, ^64^, ^65^ and unpublished data. The diagnostic rate was defined as the cumulative proportion of all patients living with MASH diagnosed with at-risk MASH at the end of a given year. Hypothetical diagnosis pathways were developed covering identification (i.e. abnormal liver enzymes), screening (e.g. with FIB-4 or the NAFLD-Fibrosis Score), and confirmatory diagnosis (e.g. with vibration controlled transient elastography, using the Enhanced Liver Fibrosis test, or liver biopsy). For each stage in the pathway, weighted average sensitivities and specificities were used depending on the proportion of different diagnostic tests. The pathways covered five healthcare settings (i.e. 1) primary care, 2) an endocrinologist/diabetologist office, 3) endocrinology/diabetology in hospital, 4) a hepatologist/gastroenterologist office, and 5) hepatology/gastroenterology in hospital) and four risk groups (i.e. a) symptom-led presentation, b) obesity, c) type 2 diabetes (T2D), and d) cardiovascular disease (CVD)). Accounting for annual F2 MASH prevalence and population growth, the number of tests required to double the diagnosis rate between 2022 and 2027 was estimated. Bottlenecks in provider capacity across healthcare settings were estimated and the location of testing adjusted accordingly. See the Supplementary Material for further details. Across the four countries, a doubling of the diagnostic rates of at-risk MASH between 2022 and 2027 was estimated to expand the number of diagnosed MASLD patients from 2.6 million to 6.1 million. Based on these estimates, the proportion of people across the different risk pools would be 54%, 19%, 18%, and 9% for the T2D, obesity, CVD, and symptom-led presentation groups, respectively. To achieve this, screening tests were estimated to grow from 2.2 million to 35.6 million and confirmatory diagnosis tests from 833,000 to 11.6 million (Fig. 3). To facilitate this expansion in testing, bottlenecks in provider capacity would need to be addressed. We estimated that the proportion of confirmatory diagnostic tests completed within liver specific settings would need to decrease from 95% in 2022 to 29% in 2027, with an increase from 1% to 27% in primary care and 4% to 34% in non-liver specialist settings, over the same time period.

## In search of a paradigm shift to bridge the MASH diagnosis gap

Paradigm shifts do not occur in a vacuum, but arise when leaps in science, technology, and innovation are coupled with changes in perception and understanding within a community. How close are we to a paradigm shift in MASH diagnoses and what are the potential catalysts, levers, and barriers to such a shift being realised?

History provides us with a useful perspective. The management of other conditions, which similar to MASH have a high prevalence, long natural history, and cause a substantial burden of morbidity and mortality, have been revolutionised over the past two decades. Routine, early screening for microalbuminuria in chronic kidney disease, dilated eye exams for retinopathy, and bone mineral density testing for osteoporotic fractures have revolutionised the management of microvascular-related diabetes complications and osteoporosis, respectively.^66^

Table 1 characterises the MASH diagnosis paradigm prior to 2024 and the opportunities for shifting the paradigm from 2024 onward across seven related domains. A confluence of factors in 2024 and expected drug approvals in many European countries in 2025 point to a near term tipping point for MASH diagnoses; nonetheless, focused and sustained efforts are needed to turn such opportunities into reality.

**Table 1:** Opportunities to shift the paradigm for MASH diagnoses.

Domain	2017–2023 paradigm	2025–2030 paradigm
MASH diagnostic tools	Focused efforts to find efficient and effective non-invasive diagnostic tools; continued reliance on liver biopsy.	Widening array of efficient and effective non-invasive tests (NITs), widely available within different healthcare settings; clear referral pathways that recommend the appropriate use of NITs, including sequential and repeat testing over time.
Recognition and awareness of MASH as a public health threat	Low awareness within the broader medical community and among at-risk persons of the importance of a timely MASH diagnosis for patient outcomes.	Growing recognition of the hepatic and extra-hepatic consequences of MASH, and inclusion of MASLD/MASH in World Health Organization (WHO) action plans and strategies for other non-communicable diseases.
Care pathways	Absent or unclearly defined care pathways within health systems inhibit the efficient movement/referral of people living with MASH between providers and services.	Well-defined care pathways in place in a growing number of health systems, often supported by digital tools and peers, helping to efficiently link people living with MASH to multidisciplinary care.
Health system policies	A near total lack of national healthcare plans and policies on MASLD and MASH, even as a part of other plans, such as for diabetes or obesity.	MASH policies and strategies in place in nearly all countries, and within regional and global normative guidance such as from WHO.
Reimbursement policies	Reimbursement of liver function tests within primary and secondary care broadly increasing, but not across all biomarker and imaging-based types; inconsistent reimbursement for treatment, including non-pharmacological interventions (NPIs).	Improving clarity over reimbursed diagnostic tests, especially in primary care and non-hepatology/gastroenterology settings; greater emphasis on defining reimbursement policies for NPIs (including digital ones) and novel MASH treatments.
MASH therapies	No approved, effective pharmacological treatment options with numerous failed late-stage drug trials; low or no implementation of NPIs.	Increasing number of therapeutic options, including the first US Food and Drug Administration approved drug for MASH treatment in 2024 and new weight-loss drugs with a beneficial impact on MASH, for which approval is expected within a year, along with digital NPIs playing an important role.
Diagnostic rate	Inconsistent emphasis on early-stage, broadly applied liver-function testing, with Scotland,^28^ and specific Hong Kong and Malaysia,^28^^,^^29^ and Camden and Islington (UK),^67^ site examples of the contrary.	Greater emphasis in many health systems for early-stage, broadly applied automated diagnoses, leading to increased diagnosis rates of MASLD, MASH, and at-risk MASH and related extra-hepatic conditions.
Prevention	Little focus on early-stage prevention (i.e. people with <F2) in healthcare settings, with attention given to the identification and management of advanced disease.	Increasingly efficient diagnostics and care models facilitate a shift to early-stage intervention in health systems and the community, globally.

Abbreviations: MASH, metabolic dysfunction-associated steatohepatitis; MASLD, metabolic dysfunction-associated steatotic liver disease.

## Conclusion

The field of hepatology has seen great advances over the past two decades in understanding the natural history of MASLD and, more recently, in diagnosing, stratifying, and treating the disease. In parallel to an improved understanding of the basic science and the condition's natural history, research and development has driven a shift from liver biopsy to NITs and led to the first approval of a MASH-specific pharmacological treatment. The next years should be characterised by concerted multistakeholder efforts encompassing an expansion of the community of practice, enhancement of health system operational readiness, and rapidly expanding disease diagnosis to provide treatment and care for the hundreds of millions of people living with MASLD globally, making a special effort for those living with at-risk MASH. We have the knowledge, tools, and opportunity to end this public health threat by 2030.

## Contributors

JVL, HEM, and CJK conceptualised the article and wrote the first draft. PNB, WA, AMA, CDB, LC, CC, KC, MMG, JM, MR, CWS, FT, VW-SW, and MN contributed to all drafts of the manuscript. JVL, HEM, and JM directly accessed and verified the underlying data. All authors had full access to all the data and approved of this version for submission to be published.

## Declaration of interests

JVL acknowledges a grant to ISGlobal to support this work from Novo Nordisk and Echosens. He also acknowledges grants to ISGlobal from AbbVie, Boehringer Ingelheim, Echosens, Gilead Sciences, Madrigal, Moderna, MSD, Novo Nordisk, Pfizer, and Roche Diagnostics, consulting fees from Echosens, GSK, Novavax, Novo Nordisk, Pfizer, and Prosciento, and payment or honoraria for lectures from AbbVie, Echosens, Gilead Sciences, Janssen, Moderna, MSD, Novo Nordisk, and Pfizer, outside of this work. PNB acknowledges consulting fees, payment or honoraria, and support for attending meetings and/or travel from Novo Nordisk, outside of this work. HEM acknowledges a grant to ISGlobal to support this work from Novo Nordisk and Echosens. He is also Director of HEM Consultancy LTD which provides consulting services within the global health and international development sectors. Through HEM Consultancy LTD he reports consulting fees from: Panorma Global, the Barcelona Institute for Global Health, the European Association for the Study of the Liver, GBCHealth, and the United Nations University-IIGH, outside of this work. WA acknowledges grants or contracts to QMUL from GSK, MSD, and Gilead, consulting fees from GSK, Novo Nordisk, Concusio, Madrigal, Janssen, UCB, Gilead, and Echosens, participation on an advisory board for GSK and Madrigal, and stock or stock options from Metadeq, outside this work. AMA acknowledges grants to her institution from Novo Nordisk, Pfizer, Target Pharma, Oncoustics, Escopics, and Siemens and consulting fees from Novo Nordisk, Madrigal, GSK, Boehringer Ingelheim, and Prosciento, outside of this work. CDB acknowledges research grants paid to his institution from Echosens, outside of this work. LC acknowledges consulting fees from Boehringer-Ingelheim, Boston Pharmaceutical, Echosens, Gilead, GSK, Madrigal, MSD, Novo Nordisk, Pfizer, Sagimet, and Siemens Healthineers, payment or honoraria from AstraZeneca, Echosens, Gilead, Inventiva, Madrigal, and Novo Nordisk, and support for attending meetings and/or travel from Novo Nordisk, outside of this work. CC acknowledges grants or contracts from Echosens, Gilead, and Novo Nordisk, consulting fees from Gilead, Novo Nordisk, AstraZeneca, Elli Lilly, E-scopics, MSD, Bayer, and Echosens, payment or honoraria from Madrigal, Novo Nordisk, and Echosens, and support for attending meetings and/or travel from AstraZeneca, outside of this work. KC has received research support towards the University of Florida as principal investigator from Boehringer Ingelheim, Echosens, Inventiva, LabCorp, Perspectum, and Target-NASH and served as a consultant for Arrowhead, 89Bio, Boehringer Ingelheim, BMS, Echosens, Eli Lilly & Co, GSK, MGGM, Novo Nordisk, Sagimet Biosciences, Terns Pharmaceuticals, and Zealand Pharma, outside of this work. MMG acknowledges payment or honoraria from Novo Nordisk, Lilly, and Echosens. CJK acknowledges a grant to ISGlobal to support this work from Novo Nordisk and Echosens. JM reports being an employee of Echosens. MR acknowledges payments to his institution from Boehringer Ingelheim and Novo Nordisk, consulting fees from Echosens and MSD, payment or honoraria from Eli Lilly, Madrigal, Novo Nordisk, and Synlab, and participation on an advisory board for AstraZeneca and Target RWE, outside of this work. CWS acknowledges speaker fees from Gilead Sciences and Sanofi, support for attending meetings and/or travel from Gilead Sciences, and being Vice-president of the Society of Liver Disease in Africa, outside of this work. FT acknowledges research funding to his institution from AstraZeneca, MSD, Gilead, and Agomab, consulting fees from AstraZeneca, Gilead, GSK, AbbVie, BMS, Ipsen, Inventiva, Pfizer, Novartis, Novo Nordisk, MSD, Madrigal, Sanofi, and Boehringer, payment or honoraria from Gilead, AbbVie, Falk, Merz, Ipsen, Sanofi, AstraZeneca, Takeda, and Boehringer, support for attending meetings and/or travel from Gilead, and participation on a data safety monitoring or advisory board for Sanofi and Pfizer, outside of this work. VW-SW acknowledges grants or contracts to his institution from Gilead Sciences, consulting fees from AbbVie, AstraZeneca, Boehringer Ingelheim, Echosens, Eli Lilly, Gilead Sciences, Intercept, Inventiva, Merck, Novo Nordisk, Pfizer, Sagimet Biosciences, TARGET PharmaSolutions, and Visirna, payment or honoraria from Abbott, AbbVie, Echosens, Gilead Sciences, Novo Nordisk, and Unilab, support for attending meetings and/or travel from AbbVie and Gilead Sciences, and being Chairman of the Specialty Board of Gastroenterology and Hepatology, Hong Kong College of Physicians, Member of the Steering Committee on the Prevention of Viral Hepatitis, Hong Kong SAR Government, and Co-founder of Illuminatio Medical Technology, outside of this work. MN acknowledges grants or contracts from Allergan, Altimmune, Akero, BI, BMS, Boston Pharma, Conatus, Corcept, Gilead, Galectin, Genfit, GSK, Kowa, Enanta, Madrigal, Lilly, Merck, Novartis, Novo Nordisk, Rivus, Shire, Takeda, Terns, Viking, and Zydus, consulting fees from Akero, Altimmune, Alligos, AstraZeneca, BI, Boston Pharma, Cytodyn, GSK, Lilly, Madrigal, Merck, Novo Nordisk, Sagimet, Terns, and Takeda, payment or honoraria from Madrigal and Novo Nordisk, and stock or stock options from Rivus Pharma, Cytodyn, Akero, and ChronWell, outside of this work.

## References

[1] Rinella M.E., Lazarus J.V., Ratziu V. (2023). A multisociety Delphi consensus statement on new fatty liver disease nomenclature. J Hepatol.

[2] Riazi K., Azhari H., Charette J.H. (2022). The prevalence and incidence of NAFLD worldwide: a systematic review and meta-analysis. Lancet Gastroenterol Hepatol.

[3] Younossi Z.M., Golabi P., Paik J.M., Henry A., Van Dongen C., Henry L. (2023). The global epidemiology of nonalcoholic fatty liver disease (NAFLD) and nonalcoholic steatohepatitis (NASH): a systematic review. Hepatology.

[4] Kleiner D.E., Brunt E.M., Van Natta M. (2005). Design and validation of a histological scoring system for nonalcoholic fatty liver disease. Hepatology.

[5] Heyens L.J.M., Busschots D., Koek G.H., Robaeys G., Francque S. (2021). Liver fibrosis in non-alcoholic fatty liver disease: from liver biopsy to non-invasive biomarkers in diagnosis and treatment. Front Med (Lausanne).

[6] Tacke F., Horn P., Wai-Sun Wong V. (2024). EASL-EASD-EASO clinical practice guidelines on the management of metabolic dysfunction-associated steatotic liver disease (MASLD). J Hepatol.

[7] Ye Q., Zou B., Yeo Y.H. (2020). Global prevalence, incidence, and outcomes of non-obese or lean non-alcoholic fatty liver disease: a systematic review and meta-analysis. Lancet Gastroenterol Hepatol.

[8] Huang D.Q., Wong V.W.S., Rinella M.E. (2025). Metabolic dysfunction-associated steatotic liver disease in adults. Nat Rev Dis Primers.

[9] Ajmera V., Cepin S., Tesfai K. (2023). A prospective study on the prevalence of NAFLD, advanced fibrosis, cirrhosis and hepatocellular carcinoma in people with type 2 diabetes. J Hepatol.

[10] Lomonaco R., Godinez Leiva E., Bril F. (2021). Advanced liver fibrosis is common in patients with type 2 diabetes followed in the outpatient setting: the need for systematic screening. Diabetes Care.

[11] Ekstedt M., Hagström H., Nasr P. (2015). Fibrosis stage is the strongest predictor for disease-specific mortality in NAFLD after up to 33 years of follow-up. Hepatology.

[12] Anstee Q.M., Targher G., Day C.P. (2013). Progression of NAFLD to diabetes mellitus, cardiovascular disease or cirrhosis. Nat Rev Gastroenterol Hepatol.

[13] Mantovani A., Petracca G., Beatrice G. (2022). Non-alcoholic fatty liver disease and increased risk of incident extrahepatic cancers: a meta-analysis of observational cohort studies. Gut.

[14] Adams L.A., Anstee Q.M., Tilg H., Targher G. (2017). Non-alcoholic fatty liver disease and its relationship with cardiovascular disease and other extrahepatic diseases. Gut.

[15] Sepanlou S., Safiri S., Bisignano C. (2020). The global, regional, and national burden of cirrhosis by cause in 195 countries and territories, 1990-2017: a systematic analysis for the global burden of disease study 2017. Lancet Gastroenterol Hepatol.

[16] Noureddin M., Vipani A., Bresee C. (2018). NASH leading cause of liver transplant in women: updated analysis of indications for liver transplant and ethnic and gender variances. Am J Gastroenterol.

[17] Miao L., Targher G., Byrne C.D., Cao Y.Y., Zheng M.H. (2024). Current status and future trends of the global burden of MASLD. Trends Endocrinol Metab.

[18] Brennan P.N., Zelber-Sagi S., Allen A.M., Dillon J.F., Lazarus J.V. (2024). Beyond a liver-gut focus: the evolution of gastroenterology and hepatology in challenging the obesity and steatotic liver disease paradigm. Gut.

[19] Brennan P.N., Kopka C.J., Agirre-Garrido L. (2025). Reviewing MAESTRO-NASH and the implications for hepatology and health systems in implementation/accessibility of Resmetirom. NPJ Gut Liver.

[20] Alexander M., Loomis A.K., Fairburn-Beech J. (2018). Real-world data reveal a diagnostic gap in non-alcoholic fatty liver disease. BMC Med.

[21] Lin H., Yip T.C., Zhang X. (2023). Age and the relative importance of liver-related deaths in nonalcoholic fatty liver disease. Hepatology.

[22] Danpanichkul P., Suparan K., Kaeosri C. (2024). Global trend of MASH-associated liver cancer: a systematic analysis from the global burden of disease 2021. Clin Gastroenterol Hepatol.

[23] Hagström H., Nasr P., Ekstedt M. (2020). Health care costs of patients with biopsy-confirmed nonalcoholic fatty liver disease are nearly twice those of matched controls. Clin Gastroenterol Hepatol.

[24] Schattenberg J.M., Lazarus J.V., Newsome P.N. (2021). Disease burden and economic impact of diagnosed non-alcoholic steatohepatitis in five European countries in 2018: a cost-of-illness analysis. Liver Int.

[25] Younossi Z.M., Blissett D., Blissett R. (2016). The economic and clinical burden of nonalcoholic fatty liver disease in the United States and Europe. Hepatology.

[26] Lazarus J.V., Mark H.E., Allen A.M. (2024). A global action agenda for turning the tide on fatty liver disease. Hepatology.

[27] Lazarus J.V., Mark H.E., Villota-Rivas M. (2022). The global NAFLD policy review and preparedness index: are countries ready to address this silent public health challenge?. J Hepatol.

[28] Dillon J.F., Miller M.H., Robinson E.M. (2019). Intelligent liver function testing (iLFT): a trial of automated diagnosis and staging of liver disease in primary care. J Hepatol.

[29] Zhang X., Yip T.C., Wong G.L. (2023). Clinical care pathway to detect advanced liver disease in patients with type 2 diabetes through automated fibrosis score calculation and electronic reminder messages: a randomised controlled trial. Gut.

[30] Harrison S.A., Bedossa P., Guy C.D. (2024). A phase 3, randomized, controlled trial of resmetirom in NASH with liver fibrosis. N Engl J Med.

[31] Sanyal A.J., Newsome P.N., Kliers I. (2025). Phase 3 trial of semaglutide in metabolic dysfunction-associated steatohepatitis. N Engl J Med.

[32] Harrison S.A., Allen A.M., Dubourg J., Noureddin M., Alkhouri N. (2023). Challenges and opportunities in NASH drug development. Nat Med.

[33] Tacke F., Puengel T., Loomba R., Friedman S.L. (2023). An integrated view of anti-inflammatory and antifibrotic targets for the treatment of NASH. J Hepatol.

[34] Mantovani A., Byrne C.D., Targher G. (2022). Efficacy of peroxisome proliferator-activated receptor agonists, glucagon-like peptide-1 receptor agonists, or sodium-glucose cotransporter-2 inhibitors for treatment of non-alcoholic fatty liver disease: a systematic review. Lancet Gastroenterol Hepatol.

[35] Sanyal A.J., Bedossa P., Fraessdorf M. (2024). A phase 2 randomized trial of survodutide in MASH and fibrosis. N Engl J Med.

[36] Loomba R., Hartman M.L., Lawitz E.J. (2024). Tirzepatide for metabolic dysfunction-associated steatohepatitis with liver fibrosis. N Engl J Med.

[37] Newsome P.N., Ambery P. (2023). Incretins (GLP-1 receptor agonists and dual/triple agonists) and the liver. J Hepatol.

[38] Sanyal A.J., Kaplan L.M., Frias J.P. (2024). Triple hormone receptor agonist retatrutide for metabolic dysfunction-associated steatotic liver disease: a randomized phase 2a trial. Nat Med.

[39] United Nations The 17 goals. UN. https://sdgs.un.org/goals.

[40] Holmer M., Lindqvist C., Petersson S. (2021). Treatment of NAFLD with intermittent calorie restriction or low-carb high-fat diet - a randomised controlled trial. JHEP Rep.

[41] Vitellius C., Desjonqueres E., Lequoy M. (2024). MASLD-related HCC: multicenter study comparing patients with and without cirrhosis. JHEP Rep.

[42] Lazarus J.V., Ivancovsky Wajcman D., Mark H.E. (2024). Opportunities and challenges following approval of resmetirom for MASH liver disease. Nat Med.

[43] Nabi O., Lacombe K., Boursier J., Mathurin P., Zins M., Serfaty L. (2020). Prevalence and risk factors of nonalcoholic fatty liver disease and advanced fibrosis in general population: the French Nationwide NASH-CO study. Gastroenterology.

[44] Abeysekera K.W.M., Valenti L., Younossi Z. (2024). Implementation of a liver health check in people with type 2 diabetes. Lancet Gastroenterol Hepatol.

[45] Allen A.M., Lazarus J.V., Alkhouri N. (2025). Global patterns of utilization of noninvasive tests for the clinical management of metabolic dysfunction-associated steatotic liver disease. Hepatol Commun.

[46] Anstee Q.M., Castera L., Loomba R. (2022). Impact of non-invasive biomarkers on hepatology practice: past, present and future. J Hepatol.

[47] Lazarus J.V., Castera L., Mark H.E. (2023). Real-world evidence on non-invasive tests and associated cut-offs used to assess fibrosis in routine clinical practice. JHEP Rep.

[48] Castera L. (2020). Non-invasive tests for liver fibrosis in NAFLD: creating pathways between primary healthcare and liver clinics. Liver Int.

[49] Boursier J., Canivet C.M., Costentin C. (2023). Impact of type 2 diabetes on the accuracy of noninvasive tests of liver fibrosis with resulting clinical implications. Clin Gastroenterol Hepatol.

[50] Gruneau L., Kechagias S., Sandström P., Ekstedt M., Henriksson M. (2023). Cost-effectiveness analysis of noninvasive tests to identify advanced fibrosis in non-alcoholic fatty liver disease. Hepatol Commun.

[51] Zhang E., Wartelle-Bladou C., Lepanto L., Lachaine J., Cloutier G., Tang A. (2015). Cost-utility analysis of nonalcoholic steatohepatitis screening. Eur Radiol.

[52] Younossi Zobair M., Paik James M., Henry L., Stepanova M., Nader F. (2025). Pharmaco-economic assessment of screening strategies for high-risk MASLD in primary care. Liver Int.

[53] Noureddin M., Jones C., Alkhouri N., Gomez E.V., Dieterich D.T., Rinella M.E. (2020). Screening for nonalcoholic fatty liver disease in persons with type 2 diabetes in the United States is cost-effective: a comprehensive cost-utility analysis. Gastroenterology.

[54] Abner S., Gillies C.L., Shabnam S. (2022). Consultation rates in people with type 2 diabetes with and without vascular complications: a retrospective analysis of 141,328 adults in England. Cardiovasc Diabetol.

[55] Lazarus J.V., Anstee Q.M., Hagström H. (2021). Defining comprehensive models of care for NAFLD. Nat Rev Gastroenterol Hepatol.

[56] Loomba R., Jain A., Diehl A.M. (2019). Validation of serum test for advanced liver fibrosis in patients with nonalcoholic steatohepatitis. Clinl Gastroenterol Hepatol.

[57] National Guideline Centre (UK) (2016). Non-Alcoholic Fatty Liver Disease: Assessment and Management.

[58] Cusi K., Isaacs S., Barb D. (2022). American association of clinical endocrinology clinical practice guideline for the diagnosis and management of nonalcoholic fatty liver disease in primary care and endocrinology clinical settings: co-sponsored by the American Association for the Study of Liver Diseases (AASLD). Endocr Pract.

[59] Kwo P.Y., Cohen S.M., Lim J.K. (2017). ACG clinical guideline: evaluation of abnormal liver chemistries. Am J Gastroenterol.

[60] Valenti L., Pelusi S., Bianco C. (2021). Definition of healthy ranges for alanine aminotransferase levels: a 2021 update. Hepatol Commun.

[61] Bohr A., Memarzadeh K. (2020). Artificial Intelligence Healthcare.

[62] Kumar Y., Koul A., Singla R., Ijaz M.F. (2023). Artificial intelligence in disease diagnosis: a systematic literature review, synthesizing framework and future research agenda. J Ambient Intell Humaniz Comput.

[63] Estes C., Razavi H., Loomba R., Younossi Z., Sanyal A.J. (2018). Modeling the epidemic of nonalcoholic fatty liver disease demonstrates an exponential increase in burden of disease. Hepatology.

[64] Younossi Z.M., Koenig A.B., Abdelatif D., Fazel Y., Henry L., Wymer M. (2016). Global epidemiology of nonalcoholic fatty liver disease-Meta-analytic assessment of prevalence, incidence, and outcomes. Hepatology.

[65] Morgan A., Hartmanis S., Tsochatzis E. (2021). Disease burden and economic impact of diagnosed non-alcoholic steatohepatitis (NASH) in the United Kingdom (UK) in 2018. Eur J Health Econ.

[66] Cusi K., Budd J., Johnson E., Shubrook J. (2024). Making sense of the nonalcoholic fatty liver disease clinical practice guidelines: what clinicians need to know. Diabetes Spectr.

[67] Srivastava A., Gailer R., Tanwar S. (2019). Prospective evaluation of a primary care referral pathway for patients with non-alcoholic fatty liver disease. J Hepatol.

