# Supplementary material for: A call for doubling the diagnostic rate of at-risk metabolic dysfunction-associated steatohepatitis
Source: Lancet Reg Health Eur. 2025 Jun 4;54:101320. doi: 10.1016/j.lanepe.2025.101320 (PMC12266185; doi:10.1016/j.lanepe.2025.101320)
Supplement: Supplementary Material [file mmc1.pdf]

Supplementary material: A call for doubling the diagnostic rate of at-risk metabolic dysfunction-associated steatohepatitis

Table of contents

Assumptions for estimating the testing requirements to double the diagnosis rate of metabolic dysfunction-associated steatohepatitis with moderate fibrosis between 2022-2027 2
Data sources and assumptions 2
Diagnostic pathway and patient pool assumptions 2
References 3

Assumptions for estimating the testing requirements to double the diagnosis rate of metabolic dysfunction-associated steatohepatitis with moderate fibrosis between 2022-2027

A patient flow forecast model–factoring both continuous and discrete events–was developed to cover 2022 to 2027, across four countries (France, Germany, the UK, and the USA), estimating the progression of the four different patient pools in the metabolic dysfunction-associated steatohepatitis (MASH) diagnosis pathway over time to estimate the resulting diagnosis rate.

Key model mechanics include the organisation of patient management across five settings considering bottlenecks and variance in improvements within each, the quantification of the annual number of tests and patients diagnosed by factoring in the mix, sequence, and relative sensitivity/specificity of tests, and forecasting growth strategies to optimise referral quality and flow while adjusting for healthcare capacity constraints.

Data sources and assumptions

A range of sources were used to input into the model, including healthcare providers (HCPs) market research surveys (primary), published scientific articles, prescription and claims data, clinical guidelines, physician and payor interviews, and healthcare system databases (e.g. National Health Service). Estimates of the MASH prevalence (all stages) was based on a triangulation of prior MASH market research (IQVIA),[1] and published work from Estes *et al.* (2018)[2] and Younossi *et al.* (2016).[3] The prevalence of at-risk MASH (i.e. MASH with ≥F2) assumed that 50% of all MASH would be at-risk MASH; this was based on prior extrapolated HCP survey data (Ipsos–unpublished).

The at-risk MASH diagnosis rates were based on informed-assumptions based on triangulation of different data sources, including Morgan *et al.* 2021[4] [for the UK], Intercept claims/laboratory data [for the USA], and Schattenberg *et al.* 2021[5] [for France, Germany, and the UK]. These sources were triangulated with prior MASH market research by IQVIA[1] and adjustments were supported by country specificities, such as the level of MASH awareness among different HCPs.

Diagnostic pathway and patient pool assumptions

The total patient pool (i.e. all people living with at-risk MASH) were allocated to one of four patient pools: symptom-led presentation, obesity, type 2 diabetes (T2D), and cardiovascular disease (CVD). A prioritisation was applied to avoid double counting of patients living with overlapping comorbidities; this prioritisation varied by management setting. In primary care settings, T2D was prioritised, followed by CVD and obesity. In liver-specialist and non-liver specialist settings, T2D was prioritised, followed by obesity and CVD.

The number of HCPs per healthcare setting and the patient load per HCP was estimated from a range of sources (Novo Country Reports (2022)–IQVIA; Novo Nordisk HCP segmentation (2022); Novo Nordisk Germany Affiliate data (2022); key informant discussions (n=20)).

Multiple data sources were used to assess the current state of the MASH diagnostic pathway across identification/screening, initial diagnosis, and confirmatory diagnosis, including the patient pool mix by healthcare setting and the proportion of specific tests used within each healthcare setting. Variations in the prevalence of the different patient pools were accounted for as part of the assessment and in the model, based on published[6] and unpublished data. Weighted average sensitivities and specificities were used for each stage in the pathway, depending on the proportion of tests used. Sensitivity and specificity for each individual test corresponds to the ability to discriminate patients living with a ≥F2 stage of fibrosis based on fixed thresholds: aspartate aminotransferase/alanine aminotransferase,[7] ultrasound,[8] FIB-4 (Echosens internal data), NAFLD-Fibrosis Score,[9] FibroScan (Internal Novo Nordisk data), and Enhanced Liver Fibrosis (Internal Novo Nordisk data).

References

1. Gores M, Scott K. Emerging from the Shadows: A New Era for NASH IQVIA, 2023.
2. Estes C, Razavi H, Loomba R, Younossi Z, Sanyal AJ. Modeling the epidemic of nonalcoholic fatty liver disease demonstrates an exponential increase in burden of disease. *Hepatology* 2018;**67**(1):123–33.
3. Younossi ZM, Koenig AB, Abdelatif D, Fazel Y, Henry L, Wymer M. Global epidemiology of nonalcoholic fatty liver disease-Meta-analytic assessment of prevalence, incidence, and outcomes. *Hepatology* 2016;**64**(1):73–84.
4. Morgan A, Hartmanis S, Tsochatzis E, et al. Disease burden and economic impact of diagnosed non-alcoholic steatohepatitis (NASH) in the United Kingdom (UK) in 2018. *Eur J Health Econ* 2021;**22**(4):505–18.
5. Schattenberg JM, Lazarus JV, Newsome PN, et al. Disease burden and economic impact of diagnosed non-alcoholic steatohepatitis in five European countries in 2018: A cost-of-illness analysis. *Liver Int* 2021;**41**(6): 1227–42.
6. Younossi ZM, Golabi P, de Avila L, et al. The global epidemiology of NAFLD and NASH in patients with type 2 diabetes: A systematic review and meta-analysis. *J Hepatol* 2019;**71**(4):793–801.
7. Valenti L, Pelusi S, Bianco C, et al. Definition of Healthy Ranges for Alanine Aminotransferase Levels: A 2021 Update. *Hepatol Commun* 2021;**5**(11):1824–32.
8. Petzold G. Role of Ultrasound Methods for the Assessment of NAFLD. *J Clin Med* 2022;**11**(15):4581.
9. Hernandez Roman J, Siddiqui MS. The role of noninvasive biomarkers in diagnosis and risk stratification in nonalcoholic fatty liver disease. *Endocrinol Diabetes Metab* 2020;**3**(4):e00127.
